# Integrating node embeddings and biological annotations for genes to predict disease-gene associations

**DOI:** 10.1186/s12918-018-0662-y

**Published:** 2018-12-31

**Authors:** Sezin Kircali Ata, Le Ou-Yang, Yuan Fang, Chee-Keong Kwoh, Min Wu, Xiao-Li Li

**Affiliations:** 1 Department of Computer Science and Engineering, Nanyang Technological University, Singapore, Singapore; 2 Department of Electronic Engineering, College of Information Engineering, Shenzhen University, China, Singapore, Singapore; 3 School of Information Systems, Singapore Management University, Singapore, Singapore; 4 Data Analytics Department, Institute for Infocomm Research, Singapore, Singapore

**Keywords:** Disease gene prediction, Node embeddings, Feature learning, Oversampling, Protein-protein interaction

## Abstract

**Background:**

Predicting disease causative genes (or simply, disease genes) has played critical roles in understanding the genetic basis of human diseases and further providing disease treatment guidelines. While various computational methods have been proposed for disease gene prediction, with the recent increasing availability of biological information for genes, it is highly motivated to leverage these valuable data sources and extract useful information for accurately predicting disease genes.

**Results:**

We present an *integrative framework* called N2VKO to predict disease genes. Firstly, we learn the node embeddings from protein-protein interaction (PPI) network for genes by adapting the well-known representation learning method *node2vec*. Secondly, we combine the learned node embeddings with various biological annotations as rich feature representation for genes, and subsequently build binary classification models for disease gene prediction. Finally, as the data for disease gene prediction is usually imbalanced (i.e. the number of the causative genes for a specific disease is much less than that of its non-causative genes), we further address this serious data imbalance issue by applying oversampling techniques for imbalance data correction to improve the prediction performance. Comprehensive experiments demonstrate that our proposed N2VKO significantly outperforms four state-of-the-art methods for disease gene prediction across seven diseases.

**Conclusions:**

In this study, we show that node embeddings learned from PPI networks work well for disease gene prediction, while integrating node embeddings with other biological annotations further improves the performance of classification models. Moreover, oversampling techniques for imbalance correction further enhances the prediction performance. In addition, the literature search of predicted disease genes also shows the effectiveness of our proposed N2VKO framework for disease gene prediction.

## Background

Studying disease-causing genes is critical towards both diagnosis and treatment for various diseases such as cancer and diabetes. Traditional linkage analysis aims to detect the chromosomal location of disease genes. However, it usually identifies a huge number of candidates. It is thus still expensive and time-consuming to identify real disease genes among these massive candidates via laboratory experiments. Therefore, various computational methods have been proposed recently to further prioritize and identify disease genes.

One of the most common strategies is to predict disease genes based on readily available protein-protein interaction (PPI) networks where nodes are proteins (gene products) and edges are the physical interactions between proteins [1–4]. Module-based methods are based on the *guilty-by-association* concept that genes within the same topological or functional modules are more likely to be associated with the same disease [5, 6]. Particularly, these methods are first designed to detect modules in PPI networks and further detect those *disease-related modules*. Meanwhile, diffusion-based methods are designed to traverse through the pathways to the known disease genes. Specifically, they initialize known disease genes as seeds and diffuse along the network through random walks. Upon convergence, the frequency of being visited for a given gene is used to rank this gene. In other words, those genes that near to the seeds are frequently visited and thus ranked higher out of all the genes. Conversely, genes placed farther from the seeds are usually less visited and scored with a lower likelihood of being associated with the disease. As a matter of fact, diffusion-based methods are widely used for prioritizing candidate disease genes, including RWR [7], RWRH [8], PRINCE [9]. As they consider both full network topology as well as the placement of the known disease genes, they are superior than the module-based methods which only consider the local neighborhood [1, 3, 10–12]. Nevertheless, diffusion-based methods focus on network propagation but usually ignore other valuable data sources (e.g., functional annotations for genes, gene expression profiles, etc), thus still less satisfactory for disease gene prediction.

Feature-based machine learning methods, on the other hand, are also widely used for disease gene prediction. They first construct representations or feature vectors for genes to describe their topological properties extracted from the PPI networks [4], e.g., degree, average distance to disease-genes, common neighbors with disease associated genes, meta-graphs [13], etc. Gene ontology annotations, gene expression profiles and other relevant information can be used as additional features for genes. Then, they train supervised learning models (e.g., SVM [14, 15]) for disease gene classification. While such features *manually* derived from PPI networks are easy to understand, we need to have good knowledge about PPI networks to manually define and extract those hand-crafted features, and we may thus lose other useful features.

Recently, automatic representation/feature learning from network/graph data through graph embedding methods [16–20] has been widely studied for graph analytics tasks. Graph embedding methods convert the graph data into a low dimensional feature space where the graph structural information and graph properties are preserved *maximally*. Specifically, firstly, they represent the graph using a set of random walk paths sampled from the graph. Secondly, deep learning models (e.g., SkipGram [21]) then work on the sampled paths to generate the node embeddings which preserve the graph properties carried by the paths. These graph embedding methods have been used for different bioinformatics applications successfully, e.g., PPI prediction [17], protein function prediction [22] and disease-pathway analysis [23].

To address the limitations of existing studies, in this paper, we propose a framework called N2VKO for disease gene prediction. Firstly, we *automatically* learn node embeddings as novel features for genes from the PPI networks by adapting a well-known graph embedding method called *node2vec* [17]. Secondly, we further integrate the node embeddings with other valuable biological information to construct comprehensive descriptors for genes and then build up machine learning models for disease gene prediction. Finally, for a specific disease, as the number of its causative genes is much less than the number of non-causative genes and thus the data is highly imbalanced, we apply oversampling techniques for imbalance correction to improve the prediction performance.

We conduct comprehensive experiments on various PPI networks. Experimental results show that integration of node embeddings and biological annotations as gene features, as well as the oversampling techniques, can improve the prediction performance. The proposed N2VKO framework is demonstrated to significantly and consistently outperform four state-of-the-arts for disease gene prediction across seven diseases. In addition, literature search also shows that N2VKO can effectively predict novel disease genes for various diseases.

## Methods

**Fig. 1:**
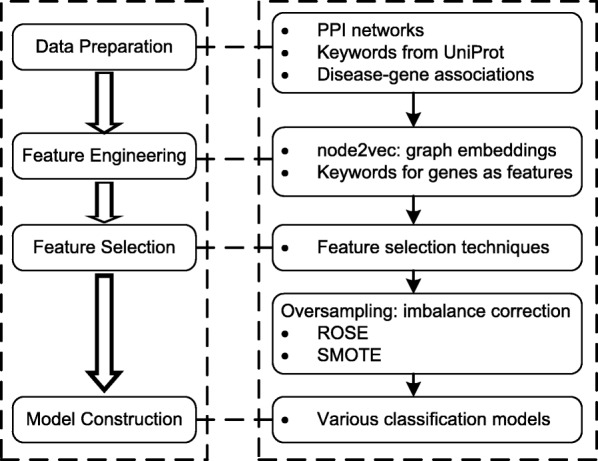
Framework of our N2VKO for predicting disease-gene associations

To better illustrate our proposed framework N2VKO, in Fig. 1, the left side shows the generic pipeline for building classification models, while the right side describes our proposed framework for predicting disease genes or discovering disease-gene associations. More specifically, after data preparation, we performed node2vec to obtain graph embeddings. We combined biological annotations with these embeddings and applied oversampling techniques before we constructed classification models. Next, we will introduce each step in N2VKO in more details.

### Data preparation

To predict novel disease-gene associations, we exploit three types of data in our study, namely, protein-protein interaction (PPI) networks, biological annotations for proteins (i.e., *Keywords*) downloaded from UniProt [24] database, and the existing disease-gene associations obtained from OMIM [25] database.

Proteins interact with each other to perform specific biological functions or tasks. Note that, we work on protein-protein interaction networks for disease gene prediction and thus we will use genes and proteins interchangeably. In a PPI network, proteins and interactions are represented by nodes and edges, respectively. Formally, a PPI network is modeled as an undirected graph *G*=(*V*,*E*), where *V* stands for the set of proteins and *E* represents the set of interactions. In particular, we work on two PPI networks, namely IntAct [26] and NCBI [27]. There are 13,063 unique proteins and 97,652 interactions in IntAct and 15,951 unique proteins and 227,004 interactions in NCBI, respectively.

To take the biological properties of individual genes into consideration, we extract the rich *Keywords* associated with each gene from the Universal Protein Resource (UniProt) database [24]. These keywords describe the various biological aspects of the genes, including their *biological processes*, *cellular components*, *molecular functions*, *coding sequence diversity*, *ligand*, *protein domains*, *post-translational modifications* (PTMs), etc. Table 1 shows the categories of the *Keywords* and corresponding examples that have been used in our study.

**Table 1:** A summary of keywords from the UniProt database

Keyword category	Examples
Biological process	*Apoptosis, Cell cycle, cAMP biosynthesis*
Cellular component	*Golgi apparatus, Vacuole, Cytoplasm*
Coding sequence diversity	*Polymorphisms, RNA-editing, Alternative splicing*
Domain	*SH2 domain, Kelch repeat, Transmembrane*
Ligand	*cAMP, S-adenosyl-L-methionine, cGMP*
Molecular function	*RNA-binding, Protein kinase inhibitor, Chromatin regulator*
Post-translational modification	*Phosphorylation, Ubiquitination, Acetylation*
Technical term	*Allosteric enzyme, Transposable element*

Disease-gene associations involve known human diseases and the human genes whose mutations causing these diseases. We extracted disease-gene associations from OMIM [25] database, which is the best-curated resource for known phenotype-genotype relationships. In this study, we aim to predict causative genes for 7 specific diseases, namely, Alzheimer’ s Disease (Al), Breast Cancer (BC), Colorectal Cancer (CC), Prostate Cancer (PC), Diabetes Mellitus (DM), Lung Cancer (LC) and Obesity (Ob). Table 2 shows the number of confirmed causative genes from OMIM for specific diseases. For example, there are 13 genes for Alzheimer in IntAct data and 47 genes for Diabetes Mellitus in NCBI data, respectively.

**Table 2:** The number of disease-genes in IntAct and NCBI data, respectively

Diseases	IntAct	NCBI
Alzheimer’ s Disease (Al)	13	14
Breast Cancer (BC)	30	32
Colorectal Cancer (CC)	34	35
Prostate Cancer (PC)	18	20
Diabetes Mellitus (DM)	37	47
Lung Cancer (LC)	18	20
Obesity (Ob)	21	33

### Learning the feature representations for genes

We first briefly introduce node2vec [17], which we adapt it to learn the feature representations for genes from the PPI networks. Then, we introduce our N2VK representations (node2vec+Keywords) which integrate both node2vec embeddings and keywords for genes.

#### node2vec

node2vec [17] is an algorithmic framework for learning feature representations for nodes in networks. Given a network, it can learn continuous feature representations for the nodes and then use these learned features for various downstream machine learning tasks, e.g., clustering, node classification and link prediction.

Inspired by the models (e.g., word2vec [21]) for natural language processing, node2vec treats a network as a “document”, where nodes are “words” and sampled walks (or paths) are “sentences”. node2vec has a flexible strategy to sample the neighborhood, which enables to alternate between breadth-first sampling strategy (BFS) and depth-first sampling (DFS) strategy.In particular, in our study, BFS consecutively extends its sampling space with immediate neighbors of the source protein in PPI network, whereas DFS extends the sampling space with sequential neighbors at increased distances from the source protein.

Consider a random walk that traversed from node *t* to node *v* and now resides at node *v*. The transition probability from node *v* to node *x*, denoted as *π*_*vx*_, is defined as *π*_*vx*_=*α*_*pq*_(*t*,*x*).*w*_*vx*_, where *w*_*vx*_ is static original edge weight between proteins *v* and *x*. In particular, the search bias function *α*_*pq*_(*t*,*x*) is defined as follows, and *p* and *q* are two parameters for node2vec.

$$\alpha_{pq}(t,x) = \left\{ \begin{array}{ll} \frac{1}{p} & \text{if} \; d_{tx} =0 \\ 1 & \text{if} \; d_{tx} =1 \\ \frac{1}{q} & \text{if} \; d_{tx} =2 \end{array}\right. $$

**Fig. 2:**
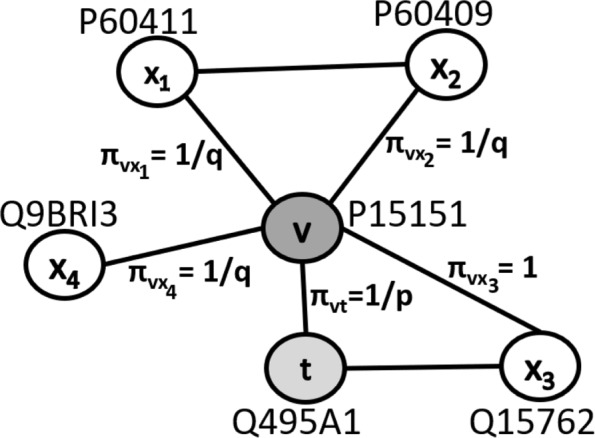
node2vec transition probabilities on a small PPI network. A random walk traversed from node *t* to node *v* and now resides at node *v*. The transition probabilities from node *v* to other proteins *x*_1_, *x*_2_, *x*_3_ and *x*_4_ are shown in this Figure

In Fig. 2, the random walk that traversed from node *t* (i.e. Q495A1) to node *v* (i.e. P15151) and now resides at node *v*. The transition probabilities from *v* to *x*_1_, *x*_2_, *x*_3_ and *x*_4_ are then determined by the shortest path distances from *t* to *x*_1_, *x*_2_, *x*_3_ and *x*_4_. For example, the shortest path distance between *t* and *x*_2_ (i.e. P60409) is 2 and *α*_*pq*_(*t*,*x*_2_) is thus $\frac {1}{q}$. Assuming the edge weight $ w_{vx_{2}} $ is 1, the transition probability $\pi _{vx_{2}}$ is then $\frac {1}{q}$ as shown in Fig. 2.

Sampled walks or paths from the PPI networks will then be fed into a single-layer neural network, which will learn vector representation for each protein. In the learning process, Skip-gram architecture [21] on network data is applied to learn the feature representation by optimizing the neighborhood preserving likelihood objective function.

#### N2VK representations

node2vec embeddings are learned from the PPI networks. Meanwhile, many databases, e.g., Gene Ontology and UniProt, provide additional information (e.g, GO annotations and keywords) for genes. To better represent genes in feature vectors for disease-gene prediction, we combine both the node2vec embeddings with the additional information for genes.

Note that our N2VKO framework is flexible to integrate node embeddings with information from different data sources. In this study, we focus on the keywords for genes obtained from UniProt, as shown in Table 1. In total, there are 554 keywords for 13,063 genes in IntAct data. As such, given a gene in IntAct, we have a 554-dimensional feature vector with binary values to describe all the keywords it has (e.g. if a gene has a certain function). Similarly, we have 567-dimensional feature vectors for genes in NCBI data. Eventually, we concatenate the node2vec embeddings with the feature vectors for keywords as our N2VK representations, i.e. node2vec embedding + keywords.

### Feature selection

After we have the N2VK representations for genes, some features may be irrelevant for predicting disease-gene associations. In addition, different feature subsets may be used to predict the disease-genes for specific diseases. Therefore, feature selection is an important step before we build the classification model for predicting disease-gene associations.

In this study, we investigate four different feature selection (FS) techniques, namely, *Earth* [28], *varImp*, *Stepwise Linear Model*, and *MRMR* (minimum redundancy – maximum relevance) [29]. We briefly introduce these FS techniques as follows.

*Earth* is a package in R [28]. It implements the non-parametric regression technique “Multivariate Adaptive Regression Splines”, commonly known as MARS. MARS is extension to the linear regression which captures nonlinearities and interactions between variables/features. In particular, the function *evimp* in *Earth* package [28] is used to estimate variable importance.

*varImp* is a function implemented in Caret package [30], which calculates variable/feature importance for regression and classification models. In particular, for linear models, it extracts variable importance based on the absolute value of the t–statistics for each model parameter.

*Stepwise Linear Model* has also been employed for feature selection. It starts with a full model with all the features, and iteratively drops a less important feature, which gives the minimum AIC (Akaike Information Criterion) value when dropped. It stops when there is no significant drop in AIC achieved and returns the remaining features as selected features.

*MRMR* [29] is a feature selection approach which aims to minimize the redundancy and maximize the relevance for the selected features. Firstly, it selects features maximally dissimilar to each other to ensure the minimum redundancy among them. Secondly, it will utilize the mutual information between the features and class labels to maximize the relevance for the features. We used the *mRMRe* package [31] in *R* in this study.

### Imbalance correction

As we can observe from the Table 2, our data for disease gene prediction is highly imbalanced. For example, we have 30 and 32 disease genes (or positive samples) for breast cancer in IntAct and NCBI, respectively. Meanwhile, IntAct and NCBI have 13,063 and 15,951 genes in total. This data imbalance issue often results in degraded performance as standard machine learning tools tend to bias towards the majority class, i.e. negative or normal genes. To address this issue, we apply two overampling techniques, namely SMOTE [32] and ROSE [33], for imbalance correction in our N2VKO.

SMOTE (Synthetic Minority Oversampling Technique) creates synthetic samples based on two parameters, namely *k* and *N*. In the feature space of minority class, SMOTE selects a sample and its *k* nearest neighbors. SMOTE further generates a data point between the selected sample and one of its *k* nearest neighbors as the synthetic data point. In addition, the parameter *N* will determine the number of synthetic data points to be generated.

ROSE (Random Over-Sampling Examples) is a R package to deal with binary classification problems for imbalanced data. In ROSE, synthetic samples are generated according to a smoothed bootstrap approach. In particular, it creates new samples from a conditional kernel density estimate of the two classes. The parameter *p* in ROSE determines the number of minority class examples in the resulting data created by ROSE.

After applying SMOTE or ROSE, we build various classification models on the balanced data, e.g., Random Forest and Support Vector Machines, for disease gene prediction. In summary, our proposed N2VKO consists of three main steps in Fig. 1. Firstly, we learn N2VK representations by integrating node2vec embeddings and keywords. Secondly, we conduct feature selection to extract subset of important features for classification. Finally, we apply oversampling techniques including SMOTE and ROSE for imbalance correction.

## Results

### Experimental setup

In our experiments, we worked on two different human PPI databases, namely IntAct [26] and NCBI [27]. We also exploited *Keywords*, i.e, biological annotations of proteins from UniProt [24] database as illustrated in Table 1. Disease labels for genes were extracted from OMIM [25] database based on phenotype entries. The number of positive examples (i.e., causative genes) for each disease is listed in Table 2.

We employed the standard metric of Area Under the ROC Curve (AUC), which is a popular and robust measure for models even upon imbalanced data and it can effectively capture the ranking effect of potential disease genes. In addition, we conduct five-fold cross-validation for each specific disease where train datasets contain 80*%* of genes while test data sets contain 20*%* genes respectively. Finally, the 5 AUC results from five-fold cross valuation were averaged to obtain the final prediction results. Note that, all our feature selection experiments were performed on the training data.

### Classification model selection

We investigated and compared 4 well-known classification models for disease gene prediction, namely, k-nearest neighbors (kNN), random forest (RF), support vector machine (SVM) and generalized linear model (GLM). kNN algorithm computes *k* nearest training set vectors for each testing sample based on Euclidean distance and the classification is performed upon majority vote. RF is an ensemble learning technique. It samples training set by sampling with replacement and uses out-of-bag data to compute an unbiased estimate of the classification error. SVM aims to find the maximum-margin hyperplane in a transformed feature space using various kernels. GLM will fit a linear equation to model the association between features and the class label.

We used different R packages for these 4 models - class package for kNN, randomforest package for RF, e1071 package for SVM and stats package for GLM. The parameters for these models are tuned to achieve their optimal performance. For example, we tuned the parameter *mtry* in RF with tuneRF function and we performed grid-based tuning for the parameter *gamma* over {10^−5^,10^−4^,10^−3^,10^−2^,0.1} and the parameter *c* over {0.1,1,10} in SVM.

**Fig. 3:**
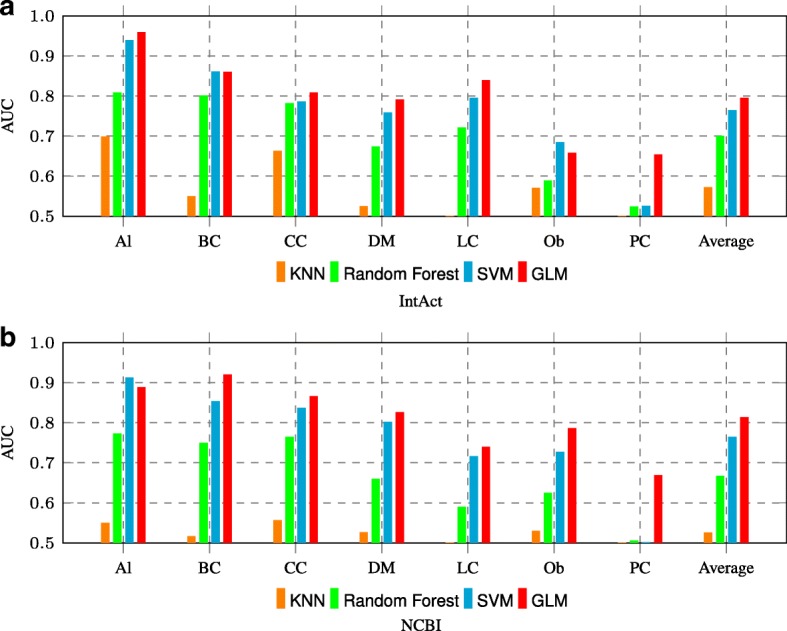
AUC comparison for various classifiers on node2vec embeddings through 7 diseases. “Average” in the last column shows the average performance of classifiers over all the seven diseases on **a** IntAct and **b** NCBI datasets

We first ran these 4 models on node2vec embeddings to predict the causative genes for the seven diseases. As shown in Fig. 3, GLM achieved the highest average AUC scores on both IntAct and NCBI. Thereafter, all the results obtained under different scenarios were based on GLM.

We performed grid-based tuning over the two parameters of node2vec, namely *p* and *q* (both *p* and *q* are selected over {0.25,0.5,1,2,4}). We chose the embedding which gives the highest AUC score and use this embedding throughout the experiments for each specific disease. For example, we set *p* as 0.25 and *q* as 2 for Alzheimer’s Disease (Al) on IntAct data. Note that different *p* and *q* values are used for different diseases.

## Discussion

### Impacts of keywords and oversampling in N2VKO

In this section, we evaluated the impacts of keywords and oversampling in our N2VKO. We compared four scenarios as follows.

- *node2vec*: node2vec embedddings were used as features without feature selection or oversampling.
- *N2VO*: Oversampling was applied node2vec embedddings without feature selection. On a specific dataset, we used the oversampling method which achieved the higher AUC performance.
- *N2VK*: Feature selection was applied on N2VK representations. On a specific dataset, we used the feature selection method (e.g., Earth, varImp, StepwiseLM and MRMR), which achieved the higher AUC performance.
- *N2VKO*: Feature selection was applied on N2VK representations. Oversampling was then applied on the selected feature subset. On a specific dataset, we used the best combination of feature selection and oversampling.

**Fig. 4:**
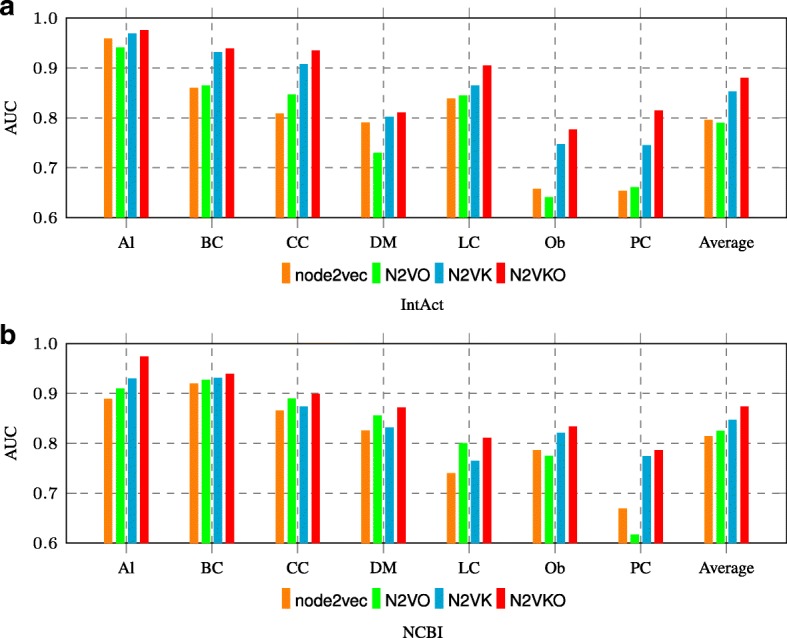
Comparison among node2vec, N2VO, N2VK and N2VKO on **a** IntAct and **b** NCBI datasets

In Fig. 4, we compared node2vec, N2VO, N2VK and N2VKO in terms of AUC on both IntAct and NCBI data sets. Similarly, the last column “Average” in Fig. 4 is the average AUC over all the seven diseases. Firstly, we observe that N2VK performs better than node2vec on both IntAct and NCBI, indicating that keywords are good complements to node embeddings for disease gene prediction. Secondly, N2VKO outperforms N2VK by 2.71% on IntAct and 2.69% on NCBI, respectively. The comparison between N2VKO and N2VK clearly demonstrates that imbalance correction by oversampling techniques indeed enhances the prediction performance.

**Fig. 5:**
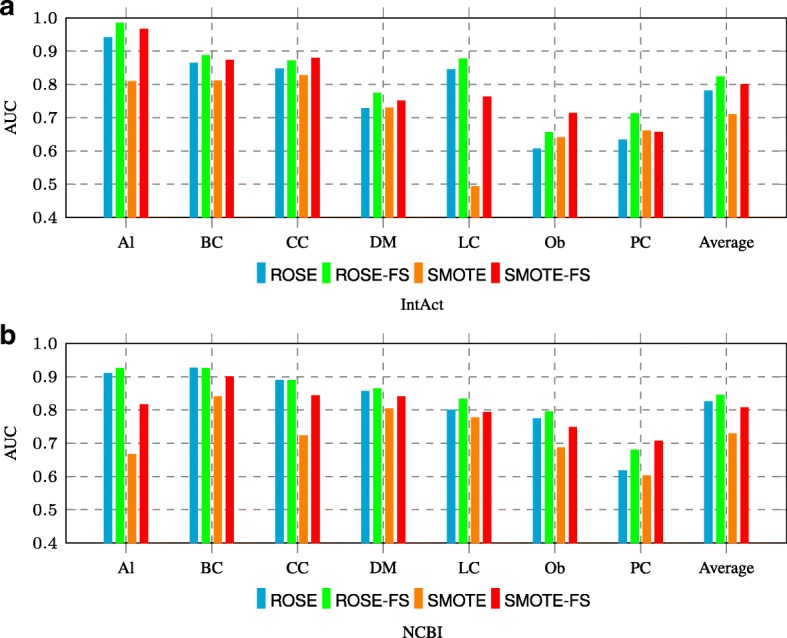
Comparison between ROSE and SMOTE on **a** IntAct and **b** NCBI datasets. **ROSE** refers to applying ROSE on node2vec embeddings, while **ROSE-FS** refers to applying ROSE after the feature selection on node2vec embeddings

In addition, N2VO and node2vec achieve comparable performance on IntAct and NCBI as shown in above Fig. 4. Note that we did not perform feature selection for node2vec embeddings in N2VO. It is usually more practical to perform feature selection before applying the oversampling techniques, and this helps to explain N2VO and node2vec achieve comparable performance. Moreover, we can clearly observe that both ROSE and SMOTE achieve better performance on FS applied features in Fig. 5. These results are consistent with the above comparison between N2VKO and N2VK in Fig. 4. In addition, ROSE achieves higher average AUC scores than SMOTE on both IntAct and NCBI, as shown in Fig. 5.

### Feature selection and importance analysis

**Fig. 6:**
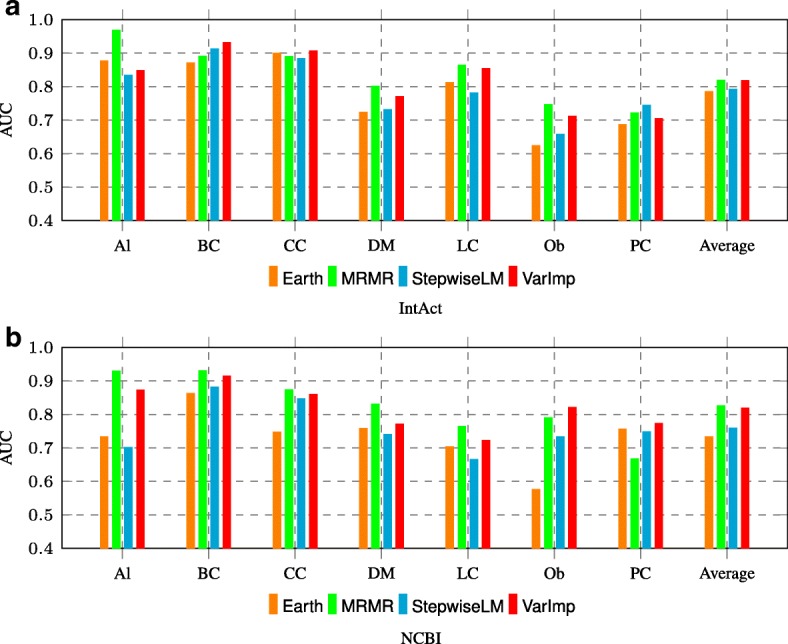
AUC comparison among 4 feature selection methods for N2VK representations on **a** IntAct and **b** NCBI datasets

In Fig. 6, we compared 4 feature selection techniques on N2VK representations. Overall MRMR and VarImp perform better than Earth and Stepwise Linear Model.

**Fig. 7:**
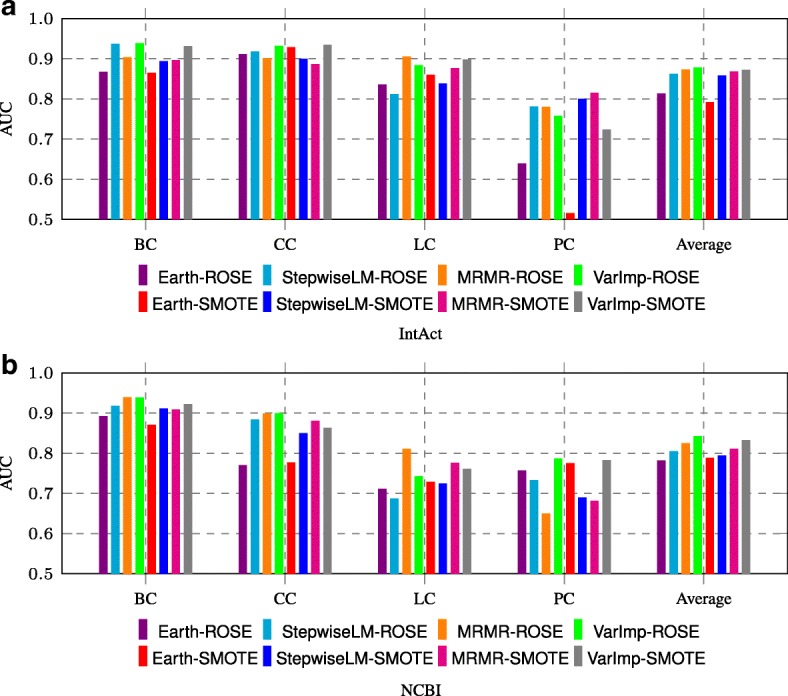
AUC comparison for various combinations of feature selection and oversampling techniques on **a** IntAct and **b** NCBI datasets. Four cancer-related diseases include *breast cancer* (BC), *colorectal cancer* (CC), *lung cancer* (LC) and *prostate cancer* (PC)

We also investigated the combinations of feature selection and oversampling to obtain the final N2VKO results. We showed the AUC scores for all the eight combinations on four cancer-related diseases in Fig. 7. For each specific disease, we selected the combination resulting in the best performance. Taking lung cancer (LC) on NCBI as example, we used the combination of MRMR and ROSE in our N2VKO framework. In addition, we can draw similar conclusions that ROSE outperforms SMOTE, and VarImp and MRMR perform better than the other two methods as shown in previous figures.

**Fig. 8:**
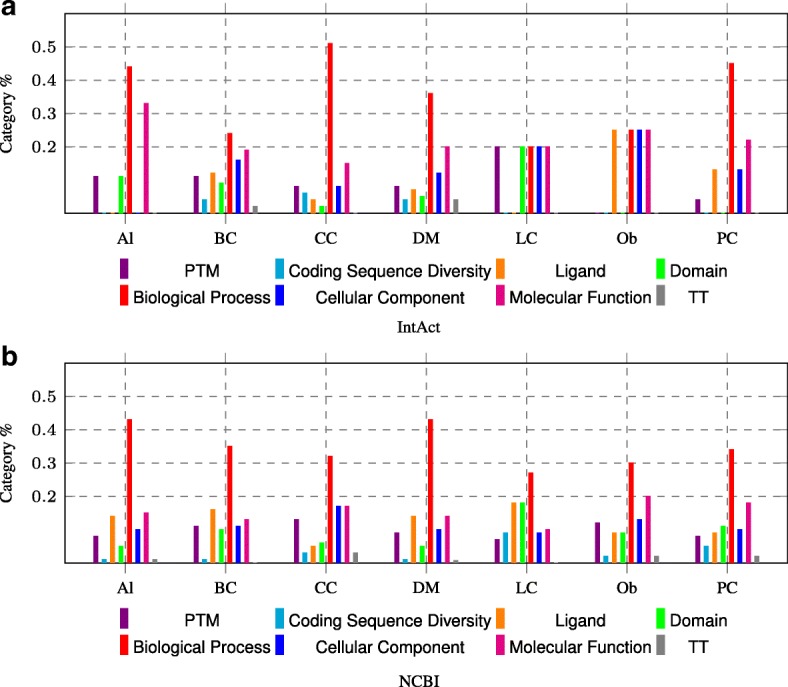
The percentages of *Keywords* categories used in N2VKO representations through 7 diseases. The percentage contribution of *Keywords* categories in N2VKO is investigated on **a** IntAct and **b** NCBI datasets

Moreover, we analyzed the selected keywords for each disease in our N2VKO and showed the percentage of their categories in Fig. 8. “Biological process” has the highest percentage and is thus the most representative category for disease gene prediction.

We further investigated the keywords selected in N2VKO to predict lung cancer genes on NCBI dataset as shown in Table 3. There are 14 keywords selected for lung cancer in Table 3, and some of these keywords are indeed important for lung cancer based on our literature search. For example, the 2 ^*nd*^ keyword in Table 3, SH3 domain, is important for lung cancer. There are approximately 300 SH3 domains encoded in the human genome, and a total of 56 human SH3 domains have been reported to be involved in the growth, proliferation, apoptosis, invasiveness, and metastasis of lung cancer [34]. In addition, the levels of “Branched-chain amino acids” such as leucine, isoleucine and valine significantly change in blood and cells of lung cancer patients as shown in [35]. Therefore, a disruption on the proteins which is responsible in synthesis of branched-chain amino acids (12^*th*^ keyword in the Table) might cause the development of lung cancer.

**Table 3:** *Keywords* in selected features for Lung Cancer on NCBI dataset

	Keyword	Category
1	Peroxisome biogenesis	Biological process
2	SH3 domain	Domain
3	Triplet repeat expansion	Coding sequence diversity
4	Serine biosynthesis	Biological process
5	Repeat	Domain
6	Antiport	Biological process
7	Dipeptidase	Molecular function
8	Cobalt	Ligand
9	Heparin-binding	Molecular function
10	Vacuole	Cellular component
11	Acetylation	PTM
12	Branched-chain amino acid biosynthesis	Biological process
13	Urea cycle	Biological process
14	Topoisomerase	Molecular function

### Comparison with state-of-the-arts

We compared our proposed N2VKO with four state-of-the-art methods for disease gene prediction, including RWR [7], RWRH [8], Catapult [14], Prodige [15] and Metagraph+ [13].

- *RWR* (Random Walk with Restart). RWR simulates iterative traversals of a walker starting from the seed genes (i.e., known disease genes) to a randomly selected neighbor in the PPI network. Different from traditional random walk, RWR is able to jump back to any seed gene with a pre-defined probability at each iteration.
- *RWRH* (Random Walk with Restart on Heterogeneous Network). RWRH prioritizes genes according to their relevance with disease genes. It combines the PPI network and phenotype network relying on the protein-phenotype associations. RWRH performs random walk with restart across inter and intra transitions in both networks.
- *Catapult*. Catapult is a Positive-Unlabeled learning model for disease gene prediction [14]. It is a supervised machine learning method that employs a biased support vector machine (SVM) upon walk-based features in a heterogeneous gene-disease network. In biased SVM, the bias assigns more penalty on false negatives than false positives to address the class imbalance issue for disease gene prediction.
- *Prodige*. Prodige is another Positive-Unlabeled learning model for disease gene prediction [15]. It builds a support vector machine model that calculates similarity scores for gene pairs. It defines two kernels for pairs of genes and pairs of phenotypes. By exploiting these two kernels, it provides the resulting kernel for gene-phenotype pairs.
- *Metagraph+*. Metagraph+ [13] provides representations for genes which include both topological features (i.e. metagraphs) and biological annotations (i.e. keywords extracted from UniProt). In our experiments, similar to N2VKO, we also applied feature selection and oversampling to predict disease genes with GLM as classification model in Metagraph+.

**Fig. 9:**
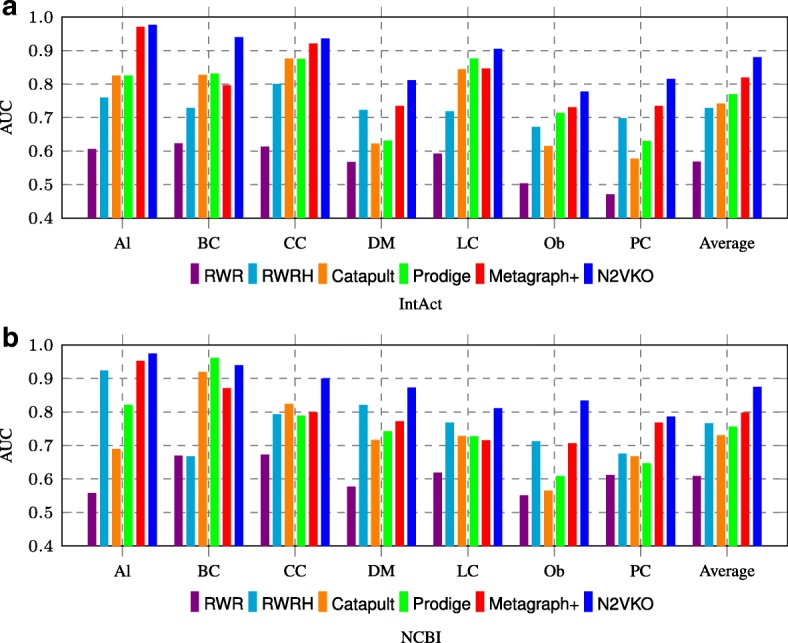
Comparison between N2VKO and various state-of-the-arts for disease gene prediction on **a** IntAct and **b** NCBI datasets

In Fig. 9, we compared AUC performances of our N2VKO with state-of-the-art methods in this field. N2VKO on IntAct data outperforms RWR, RWRH, Catapult, Prodige and Metagraph+ by 31*%*, 15*%*, 14*%*, 11*%*, and 6*%* respectively. Similarly, on NCBI data, N2VKO outperforms by 27*%*, 11*%*, 14*%*, 12*%*, and 7*%*. Clearly, N2VKO performs better than these existing algorithms for disease gene prediction on both IntAct and NCBI data.

**Fig. 10:**
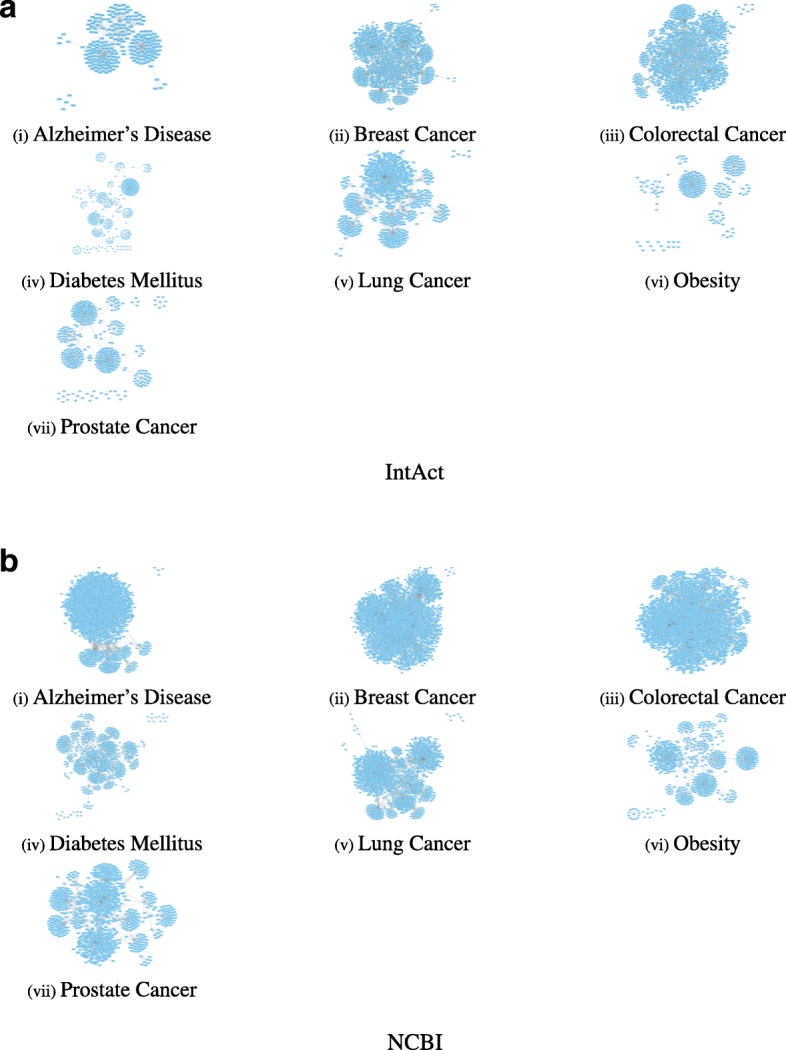
One-hop sub-networks for various diseases on on **a** IntAct and **b** NCBI datasets

We observed that N2VKO achieved low performance on obesity (Ob.) and prostate cancer (PC) data. To investigate the reasons behind, we first visualized the interactions for all the disease genes in PPI networks. For a specific disease, we constructed a sub-network where all the nodes are either the genes for this disease or their direct neighbours (one-hop neighbours). Figure 10 shows the sub-networks for all the 7 diseases and we can roughly observe that Ob and PC sub-networks are very sparse.

**Fig. 11:**
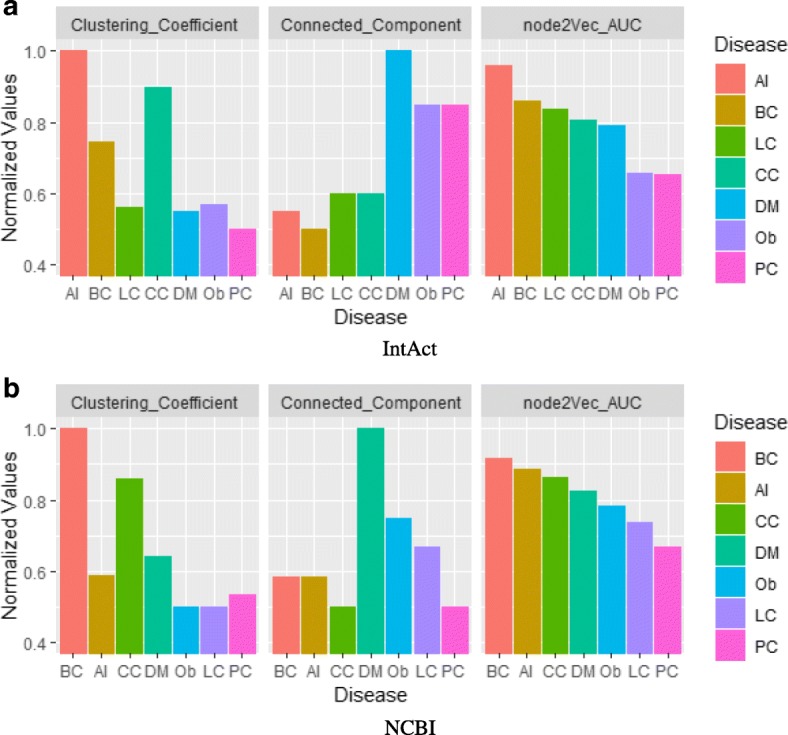
Connected component and clustering coefficient analysis for one-hop disease sub-networks on **a** IntAct and **b** NCBI datasets

Moreover, we calculated the clustering coefficient scores and counted the number of connected components for these sub-networks as shown in Fig. 11. We normalized both the clustering coefficient scores and the number of connected components into the range [0.5, 1]. For DM, PC and Ob on IntAct, they have more connected components and smaller clustering coefficient scores than others, and they achieve low AUC. On NCBI, PC, LC and Ob have much smaller clustering coefficient scores than others and they achieve lower performance. Therefore, it is clear that high sparsity (low clustering coefficient scores) will result in low prediction performance. High sparsity prohibits the influence propagation in the network, and RWR (Random Walk with Restart) thus achieves extremely low performance for PC and Ob on IntAct and Ob on NCBI as shown in Fig. 9. Hence, it is also reasonable that node2vec, a random walk based graph embedding algorithm, achieves poor results in those sparse networks.

### Case studies

We conducted further analysis for the disease genes predicted by our N2VKO. In particular, we prioritized the candidate genes based on their prediction scores. For each top-ranked gene, we searched in DisGeNET database [36] for the PubMed records reporting the diseases associated with this gene.

In Table 4, we showed the genes predicted by N2VKO on IntAct, which are ranked as top 10 predictions and also have at least 10 PubMed records supporting our predictions. The column “#PMs” shows the number of PubMed records which support the predicted disease-gene associations. As shown in Table 4, STK11 is a candidate gene for breast cancer (BC) with the highest prediction score. There are also 23 PubMed publications reporting the association between STK11 with breast cancer and “Breast Carnicoma” is the most frequent label in these publications. In addition, RAD51C’s rank is 8 and it has 14 supporting PubMed records. Similarly, genes on NCBI with top 10 prediction scores and at least 10 PubMed supports are listed in Table 5. Both Tables 4 and 5 demonstrate that our N2VKO is capable of predicting novel genes for different diseases effectively.

**Table 4:** Predicted disease genes on IntAct with top 10 prediction scores and at least 10 publications in PubMed records

Disease	Rank	Gene symbol	*#* PMs	Related diseases in PubMed records
Breast Cancer (BC)	1	STK11	23	Breast Carcinoma
	6	AKT2	11	Malignant neoplasm of breast
	7	PGR	465	Malignant neoplasm of breast, Breast Carcinoma
	8	RAD51C	14	Malignant neoplasm of breast, Hereditary Breast and Ovarian Cancer Syndrome
Diabetes Mellitus (DM)	1	AKT1	15	Diabetes Mellitus Non-Insulin-Dependent, Diabetes Mellitus
Lung Cancer (LC)	1	TNFSF10	39	Carcinoma of lung, Non-Small Cell Lung Carcinoma
	3	AURKA	13	Malignant neoplasm of lung
	4	IL24	20	Lung Neoplasms, Carcinoma of lung

**Table 5:** Predicted disease genes on NCBI with top 10 prediction scores and at least 10 publications in PubMed records

Disease	Rank	Gene symbol	*#* PMs	Related diseases in PubMed records
Breast Cancer (BC)	2	IL24	16	Breast Carcinoma
	3	NAT2	36	
	4	NBN	37	
Colorectal Cancer (CC)	6	MRE11	11	Colorectal Cancer, Malignant tumor of colon
Diabetes Mellitus (DM)	2	EIF2AK3	14	Diabetes Mellitus Insulin-Dependent, Diabetes
Lung Cancer (LC)	3	HRAS	32	Lung Neoplasms, Malignant neoplasm of lung
Obesity (Ob)	2	NPY	65	Obesity
	4	SREBF1	21	
Prostate Cancer (PC)	1	ERBB2	87	Prostate carcinoma, Malignant neoplasm of prostate

## Conclusions

Identification of disease-related genes by means of computational approach provides cost-effective techniques for the diagnosis and treatment of many diseases. In this study, we proposed a framework N2VKO to exploit human PPI networks and biological aspects of genes and predict disease-gene associations. In particular, we learned node embeddings from the PPI networks and extracted the keywords from UniProt as features for genes to build various classification models. Considering the data is highly imbalanced, we also applied oversampling techniques for imbalance correction. Comprehensive experimental results show that integration of node embeddings and keywords as gene features, as well as the oversampling techniques, can effectively improve the prediction performance. The proposed N2VKO significantly outperforms the state-of-the-arts including RWR, RWRH, Catapult, Prodige and Metagraph+ for disease gene prediction across 7 diseases. Literature search also demonstrates that N2VKO can effectively predict novel genes for various diseases.

## Abbreviations

Al: Alzheimer’s disease
BC: Breast cancer
BFS: Breadth-first sampling
CC: Colorectal cancer
DFS: Depth-first sampling
DM: Diabetes mellitus
FS: Feature selection
GO: Gene ontology
LC: Lung cancer
MRMR: Minimum redundancy - maximum relevance
Ob: Obesity
OMIM: Online mendelian inheritance in man
PC: Prostate cancer
PPI: Protein-protein interaction
PTM: Post-translational modification
RWR: Random walk with restart [7]
RWRH: Random walk with restart on heteregeneous network [8]
TT: Technical term
